# Discovering Inflammation in Atherosclerosis: Insights from Pathogenic Pathways to Clinical Practice

**DOI:** 10.3390/ijms25116016

**Published:** 2024-05-30

**Authors:** Cristina Madaudo, Giuseppe Coppola, Antonio Luca Maria Parlati, Egle Corrado

**Affiliations:** 1 Department of Health Promotion, Mother and Child Care, Internal Medicine and Medical Specialties, Cardiology Unit, University of Palermo, University Hospital P. Giaccone, 90127 Palermo, Italy; cristina.madaudo@unipa.it (C.M.);; 2 Department of Advanced Biomedical Sciences, University of Naples Federico II, 80131 Naples, Italy

**Keywords:** atherosclerosis, inflammation, cardiovascular disease, cerebrovascular disease, preventive cardiology, carotid atherosclerosis

## Abstract

This comprehensive review explores the various scenarios of atherosclerosis, a systemic and chronic arterial disease that underlies most cardiovascular disorders. Starting from an overview of its insidious development, often asymptomatic until it reaches advanced stages, the review delves into the pathophysiological evolution of atherosclerotic lesions, highlighting the central role of inflammation. Insights into clinical manifestations, including heart attacks and strokes, highlight the disease’s significant burden on global health. Emphasis is placed on carotid atherosclerosis, clarifying its epidemiology, clinical implications, and association with cognitive decline. Prevention strategies, lifestyle modifications, risk factor management, and nuanced antithrombotic treatment considerations are critical to managing cardiovascular complications, thus addressing a crucial aspect of cardiovascular health.

## 1. Introduction

Cardiovascular diseases (CVD) are the leading cause of disability and mortality worldwide [1,2,3,4], and atherosclerosis is the primary pathological process involved in most of them. Atherosclerosis is a systemic and chronic pathological alteration of the arterial wall layers that begins early in life and remains latent and asymptomatic for a long time before progressing to advanced stages [5,6]. Atherosclerosis is a multidistrict pathological process, widely studied in recent decades, which tends to develop over time in an entirely asymptomatic manner, manifesting symptoms in an advanced stage, difficult to manage from a clinical point of view, with even fatal events (acute myocardial infarction, ischemic cerebral stroke). The first structural change in the arterial wall during atherogenesis is an increase in the thickness of the intima and media layers associated with reduced elasticity and increased stiffness [7]. The systemic inflammatory component that characterizes atherosclerosis is undoubtedly one of the main aspects, as it can damage the body’s vessels at the level of different organs with various clinical manifestations. Identifying and correcting cardiovascular (CV) risk factors and implementing primary prevention is an excellent strategy. Even more effective would be the approach of identifying and monitoring natural early markers of “preclinical atherosclerosis”, which would allow the identification of the patients most at risk of developing atherosclerotic disease and its clinical manifestations.

## 2. Atherosclerosis

### 2.1. Definition

At the beginning of the 19th century, Karl von Rokitansky described for the first time the phenomenon of thickening of the walls of arterial vessels, associated with the deposition of substances on the endoluminal side, which he identified as the cause of spontaneous obliteration of the arteries [8]. The first classification of atherosclerotic lesions was instead carried out by Ludwig Aschoff, who was the first to describe the pathophysiological mechanism that leads to the formation of the actual atherosclerotic plaque composed of two components, lipid and fibrotic [9,10,11]. In the 1950s, the WHO introduced the concept of “complicated atherosclerotic plaque” to indicate lesions morphologically characterized by hematomas, thrombi, ulcerations, hemorrhages, and fissures [8]. In 1994, the American Heart Association (AHA) proposed a classification of atherosclerotic lesions, which divided them into eight categories of structurally distinct lesions, considering the pathophysiological evolution of the atherosclerotic process [12]. A real step forward occurred with the modern classification developed from 2000 by Virmani et al. [12,13] which overcame the limitation of the lack of unique morphological elements capable of identifying clinically relevant lesions. This classification divided atherosclerotic lesions into four main types: non-progressive intimal lesions, progressive atherosclerotic lesions, lesions with thrombi, and lesions with ruptures and healing outcomes [12]. The concept has evolved, and atherosclerosis has been the protagonist of various studies for some time now. Therefore, we can now define atherosclerosis as a chronic inflammatory disease of the arteries and which is the underlying cause of approximately 50% of all deaths in Westernized society. It is primarily a lipid-driven process initiated by accumulating low-density lipoprotein and residual lipoprotein particles and an active inflammatory process in focal areas of the arteries. It is considered a primary cause of atherosclerotic cardiovascular disease (ASCVD), resulting in heart attack, stroke, and peripheral arterial disease [14,15]. Increased intima-media thickness (IMT) is defined as a thickness greater than 0.9 mm. The relationship between thickness and cardiovascular risk is proportional, and in adults and older people, a high IMT is associated with a higher CV risk [16,17].

### 2.2. Preclinical Atherosclerosis

Being aware of the anatomopathological, cytokine, cellular, and ultrastructural aspects of the lesions is fundamental for the study of the pathogenesis of the atherosclerotic disease, as well as for the identification of clinically relevant lesions, for the stratification of the patient’s global CV risk, and finally to be able to develop adequate prevention and treatment strategies. The condition known as adaptive intimal thickening, as described by Virmani et al. [12,13], can be found in all arteries that, due to anatomical and hemodynamic reasons, are continually predisposed to pressure conditions that could lead to the development of atherosclerotic lesions over time (coronary arteries, carotids, aorta, iliac, femoral, etc.). Although it is the starting point, this lesion is considered a “physiological response” to blood flow rather than a true phase of the atherosclerotic process. Adaptive intimal thickening is already detectable in infant autopsy findings and can increase with age. It is identified at the cellular level by accumulating macrophages rich in lipid vesicles (foam cells) isolated or in small groups in the intima layer [8,13]. The first step of the atherosclerotic process is identified by the appearance of intimal xanthoma (lipid streak), characterized by the presence in the intima of groups of foam macrophages associated with smooth muscle cells filled with lipids. It has been demonstrated that this type of lesion can progress over time with adequate pathophysiological stimulus, such as inflammation or wall stress [13]. Pathological intimal thickening, on the other hand, represents the earliest atherosclerotic lesion with a tendency to evolve towards an atherosclerotic plaque. Changes in the extracellular matrix, an inflammatory cytokine microenvironment, and accumulation in the intima of cellular residues from the apoptosis of foam smooth muscle cells characterize it [12,13]. These phases identify the cellular and pathophysiological characteristics of the onset of the atherosclerotic process. They would find their meaning from a medical and patient management point of view in the concept of “preclinical atherosclerosis”, a phase in which atherogenesis, while altering the anatomy of the vessel, does not compromise its functionality and is clinically silent. [18,19,20,21]. Understanding the pathogenesis of atherosclerotic disease helps to understand that preclinical atherosclerosis represents only a first phase that can evolve to develop the classic clinical manifestations of cardiovascular diseases [22,23,24,25,26]. These essential concepts help us understand why it is crucial to investigate these early stages of the disease when approaching patients suffering from atherosclerotic disease. In clinical practice, the main aim is early diagnosis to obtain the best therapy and prognosis. 

## 3. Inflammation in Atherosclerosis

### 3.1. The Historical View of Inflammation in Atherosclerosis

Inflammation represents one of the main pathways in the genesis of the atherosclerotic process leading to the formation of atherosclerotic plaque and its consequent clinical events. The two fundamental aspects enjoying the greatest support from experimental data and characterizing the formation of atherosclerotic plaque are represented by the so-called “response-to-injury” theory closely related to the “inflammatory theory”. Ross R et al. [27] identified endothelial damage as the “primum movens” of the atherosclerosis process, followed by endothelial disruption and smooth muscle cell proliferation in response to circulating growth factors, particularly platelet-derived growth factor (PDGF). The endothelial lining of blood vessels is not just a set of cells acting as an inert barrier between blood and surrounding tissues but a true organ with autocrine–paracrine and endocrine functions, capable of secreting numerous chemical mediators essential for maintaining vascular function homeostasis in response to a wide variety of signals [28]. In the presence of cardiovascular risk factors, the protective role of the endothelium seems to be altered, leading to endothelial dysfunction characterized by compromised vasomotor response to major endothelium-dependent vasodilator stimuli and pro-inflammatory and pro-coagulant activity of the endothelium itself. One of the key elements of this condition is the reduced bioavailability of NO [29]. Alteration of the endothelial membrane induces an inflammatory response with consequent expression of adhesion proteins on the endothelium, such as VCAM-1 and P-selectin, which bind leukocytes such as T lymphocytes and monocytes. Following binding, these cells are internalized into the sub-intimal layer where, due to a cytokine-rich inflammatory microenvironment, they assume the appearance and characteristics of macrophages [18]. The same cytokine-rich microenvironment induces the migration of smooth muscle cells from the media and intima and the uptake of large amounts of lipids by smooth muscle cells and macrophages, leading to the formation of lipid streaks [18]. Subsequent proliferation of smooth muscle cells, collagen deposition, and other ECM components, along with the ongoing inflammatory response, lead to progressive remodeling of the vessel’s media and adventitia and the growth of the lipid streak, which quickly leads to the formation of the atherosclerotic plaque consisting of the atheroma or lipid core and an overlying fibrous cap separating the atheroma from the vascular lumen [30]. Over time, the lipid core increases in volume due to continuous lipid accumulation and apoptosis of foam cells, while the fibrous cap tends to become thinner due to proteolysis mechanisms by collagenases and metalloproteinases. This leads to the so-called “vulnerable plaque” potentially at risk of rupture [31]. When this plaque, rich in lipids, is exposed to the bloodstream, it activates tissue factor (TF), initiating the formation of a fibrin monolayer through the coagulation cascade as well as the recruitment of circulating platelets and leucocytes. The connection between the plaque components, platelet receptors, and coagulation factor triggers platelet activation and aggregation, culminating in the development of a thrombus on top of the plaque that obstructs the arterial passage leading to ischemic events, depending on the area of interest, such as acute myocardial infarction, ischemic stroke, or acute peripheral ischemia [32].

### 3.2. The New Perspective of Inflammation in Atherosclerosis

Nowadays, with modern techniques like single-cell RNA sequencing (RNA-seq) and mass cytometry, it is possible to analyze the diverse phenotypes of cells found in atherosclerosis. As has long been known, macrophages are among the most represented cell types in atherosclerosis. In a recent meta-analysis, Zernecke et al., analyzing the cell composition of atherosclerotic plaque in mouse aortas, identified five subsets of macrophages, namely, resident-like, foamy Trem 2, inflammatory, interferon-inducible (IFNIC), and cavity macrophages [33]. Each of these macrophage classes expresses different genes and performs different functions. The resident-like, in addition to genes derived from the yolk sac, such as lymphatic vessel endothelial hyaluronan receptor 1 (Lyve 1) and mannose receptor C-type (Mrc 1) of uncertain role, express platelet factor 4 (PF4), a protein that plays a key role in the platelet/macrophage connection. The foamy Trem 2 macrophages have significant expression of genes implicated in lipid metabolism, cholesterol efflux, and lysosome function, but a low expression of inflammatory-response genes [34,35,36]. In contrast with foamy Trem 2, the inflammatory macrophages have a strong proinflammatory gene profile thanks to the release of several chemokines, such as CCL3 and CCL4, and cytokines, such as TNF-α. The IFNIC macrophages are a specialized group that exhibits distinct traits associated with a type 1 interferon reaction. Although their role is unclear due to the type 1 interferon signaling in promoting atherosclerosis, this cell cluster may be implicated in advancing the disease. The cavity macrophages represent a recent cluster whose role is still unclear. In addition to macrophages, T cells play a key role in forming atherosclerotic lesions. Fernandez et al., revealed that T cells constitute approximately 65% of leukocytes in human carotid endarterectomy, underscoring their prominence in atherosclerosis [37]. Some studies support the concept of atherosclerosis as a true autoimmune T-cell disease affecting the arterial wall where the autoimmune component of atherosclerosis is driven by autoreactive CD4+ T cells [38,39]. These studies showed the highest degree of plaque-specific clonal expansion in CD4+ effector T cells in single-cell T-cell receptor sequencing (scTCR-seq) on human carotid artery plaque and peripheral blood mononuclear cell samples [38]. Several T-cell subsets can be detected within atherosclerotic lesions, with CD4 subsets, such as TH1, TH2, TFH, Treg, and TH17, being prominent [40]. Among these, the TH1 subset emerges as the predominant subset in human and murine atherosclerotic plaques. Chen et al. discovered that TH1 cells led to inflammation and plaque instability, while their genetic deficiency attenuated atherosclerosis in hyperlipidemic murine models [41,42]. Following TH1, TH2 cells constitute a substantial T-cell subgroup within atherosclerotic lesions. The functional role of TH2 cells is still controversial due to the release of some interleukins, such as IL-4, that exhibit pro-atherogenic properties in murine models and other interleukins, such as IL-13, that exhibit anti-atherogenic properties [43,44]. T follicular helper (TFH) cells release many interleukins, including IL-21, crucial for B-cell activation and differentiation and implicated in vascular dysfunction and hypertension [45]. This could suggest a potential contributory link between TFH cells and atherogenic progression. Treg cells, characterized by the secretion of atheroprotective cytokines like IL-10, play a crucial role in regulating T-cell-mediated immune responses [46]. Depletion of Treg cells exacerbates atherosclerosis in murine models, affirming their atheroprotective role [47]. TH 17 cells are activated primarily by their main cytokine, IL-17. Butcher et al. discovered that IL-17 and its receptor IL-17RA activate an axis that increases the inflammation and instability of the atherogenesis process through the continuous recruitment of neutrophils and monocytes [48]. In addition to CD4 T cells, CD8 T cells are abundantly present within atherosclerotic lesions, even though their role remains ambiguous. Kyaw et al., demonstrated that CD8 T cells depletion in murine models ameliorated atherosclerosis by reducing lipid and macrophage accumulation, apoptosis, and necrotic cores, suggesting a potential pro-atherogenic role [49]. Conversely, van Duijn et al.l., discovered that in advanced atherosclerosis, CD8 T cells depletion in mice led to larger and more unstable plaques, accompanied by reduced collagen content and increased TH1 and macrophage infiltration, implying a protective role for CD8 T cells in this context [50]. In addition to T cells, B cells also perform a significant role in the atherosclerotic process. B cells can be detected in both healthy and atherosclerotic arteries, which perform protective and pro-atherogenic effects according to a subtype of B cells. B1 and marginal-zone B cells are thought to provide protection against atherosclerosis due to the production of IgM antibodies that bind oxidized low-density lipoprotein and apoptotic cells. In contrast, follicular B cells and innate response activator B cells stimulating the Th1 phenotype promote atherosclerosis [51,52,53].

Closely associated with cell types in atherosclerosis are chemokines, small signaling proteins that play a key role in regulating inflammatory cell trafficking and the genesis and growth of atherosclerosis [54]. Many chemokines appear to be involved in atherosclerosis formation and growth, with some pathways extensively researched and others still under exploration. Recent preclinical studies have demonstrated that the CCL2-CCR2 pathway is a key driver of atherosclerosis. Deleting CCL2 or CCR2 in murine models reduced atherosclerotic development and diminished macrophage deposit within plaques, while overexpression of CCL2 enhanced atherosclerosis [55,56]. A recent meta-analysis corroborated these findings by confirming that pharmacological blockade of the CCL2 or CCR2 pathway led to a significant reduction in the formation of atherosclerotic lesions while simultaneously increasing the number of smooth muscle cells and collagen deposition, resulting in a final effect of plaque stabilization [57]. These data are further supported by examining atherosclerotic lesions stemming from 1199 subjects who underwent endarterectomy. In this analysis, CCL2 levels were markedly correlated with markers of plaque vulnerability, such as enlarged lipid core and decreased collagen deposition [58]. Furthermore, a recent Mendelian randomization study has demonstrated that a genetic predisposition to higher circulating levels of CCL2 is linked to an increased risk of stroke, CAD, and myocardial infarction [59]. The CXCL12-CXCR4 axis is another pathway that plays a crucial role. In a recent study, Doring et al. demonstrated how the deletion of CXCR4 in endothelial or smooth muscle cells of arteries significantly accelerated the genesis and development of atherosclerotic plaques in hyperlipidemic mice. The CXCL12-CXCR4 axis promotes endothelial barrier function through VE-cadherin expression and the stabilization of junctional VE-cadherin complexes in arterial endothelial cells. At the same time, its deficiency led to arterial leakage and the recruitment of inflammatory leukocytes during atherogenesis. In arterial smooth muscle cells, CXCR4 supports normal contractile responses. At the same time, its deficiency causes an impairment of vascular response and cholesterol efflux as well as promoting the occurrence of inflammatory macrophages in atherosclerotic plaques [60]. Furthermore, according to Zernecke et al., the continuous inhibition of systemic CXCR4 through its antagonist AMD3465 led to increased levels of neutrophils, resulting in a higher presence of them within atherosclerotic lesions, promoting growth and instability [61]. Vascular CXCR4 limits atherosclerosis by maintaining arterial integrity, preserving endothelial barrier function, and cell-specific enhancement of CXCR4 in vascular cells or B cells was able to therapeutically reduce atherosclerosis in mice [60,62,63]. Among chemokines, an important role is played by CXCL9, CXCL10, and CXCL11, which are released by different cell phenotypes and share the same receptor CXCR3. CXCL10, in particular, is released by activated T cells and helps retain T cells within the lesions. In ApoE−/− CXCL10−/− murine models fed a Western diet, reduced atherogenesis and an increased number of Treg cells in the lesions were observed, and antibody inhibition of CXCL10 in ApoE−/− mice led to enhanced plaque stability [64]. Consistent with these findings, CXCL10 is linked to developing instable plaques. 

In addition to the data mentioned above, in recent years, an increasing number of studies have suggested that the nervous, the immune, and the cardiovascular systems engage in intricate communication with each other in a system called neuroimmune cardiovascular interfaces (NICIs). Specifically, Mohanta et al. discovered that in murine models, the adventitia of large arteries contained a lot of nerve fibers that formed tight connections with both immune cells and media smooth muscle cells [65]. In mice, NICIs established circuits connecting atherosclerotic arteries to the brain, which involved sensory neurons of dorsal root ganglia reaching the central nervous system and efferent sympathetic and vagal fibers reaching the adventitia of arteries [66]. These data are confirmed by the evidence that surgically removing the sympathetic celiac ganglia from the abdominal portion of the aorta in mice reduced the severity of atherosclerosis in this area [65]. Previously, some studies reported the presence of aggregates of immune cells defined as artery tertiary lymphoid organs (ATLOs) in adventitia segments of atherosclerotic arteries, but not in the adventitia of normal arteries [67,68]. With the recent discovery of NICIs, this network, initially believed to be bidirectional due to the presence of ATLOs, is a network in which three systems communicate and strongly influence each other [65]. This consideration is further confirmed by the evidence that acute mental stress as well as sleep disorders dramatically increase inflammatory response and plaque development and vulnerability [69,70]. Nowadays, several studies are ongoing to better understand these tight connections and identify new potential therapeutic targets to incorporate into the treatment of atherosclerotic disease. In addition to the research on NICIs, clonal hematopoiesis (CH) is increasingly emerging as a significant factor in the pathogenesis of atherosclerosis. CH is a condition whose incidence increases with age and arises from the acquisition of somatic mutations in hematopoietic stem cells (HSC) that, when they occur in key regulatory genes, can provide a selective advantage to this cell, resulting in clonal expansion. The connection between CH and atherosclerosis has been primarily identified for somatic mutations in TET 2 (Tet methylcytosine dioxygenase 2) and JAK 2 (Janus kinase 2) genes. TET 2 is an epigenetic regulator gene often mutated in CH. Fuster et al. discovered that macrophages with TET2 loss of function exhibited increased expression of IL-1β, its precursor pro-IL-1β, and components associated with the NLRP3 inflammasome [71]. Il-1 exhibit a strong proatherogenic effect through stimulation of adhesion molecules, such as ICAM-1, that recruit leukocytes, induction of chemokines, such as CCL-2, that recruit mononuclear phagocytes, and stimulation of SMC proliferation [72,73]. The implication of inflammasome products in the atherosclerotic process is further supported by the observation that treatment with NLRP3 inhibitors reduced the size of atherosclerotic plaques [71]. JAK 2 is a tyrosine kinase involved in many cellular processes, including cell growth and differentiation. The best-known mutation in JAK 2, JAK 2V617F, is a gain of function that constitutively triggers the JAK/STAT pathway. Fidler et al. discovered that JAK 2V617F induced alterations in cellular metabolism and proliferation, resulting in DNA oxidative damage and activation of the AIM2 inflammasome with the production of IL-1β that further enhanced cell proliferation, including macrophages. These cascading events contributed to an overall rise in the presence of inflammatory macrophages within atherosclerotic lesions as well as an increase in necrotic core formation that contributed to plaque instability [74]. In addition to TET2 and JAK2, somatic mutation in other regulatory genes, such as DNMT3A, TP53, and ASXL1, in CH, and their potential connection with the development of the atherosclerotic process, are subjects of daily study. Inflammation is a key factor in the development and progression of ASCVD. While chronic inflammation contributes to plaque formation and instability, leading to adverse cardiovascular events, it also plays crucial beneficial roles. Inflammation is essential for cardiac repair, promoting the healing process after myocardial injury by clearing dead cells and facilitating tissue regeneration. Additionally, it serves as a critical component of the body’s host defense mechanisms, protecting against infections and other external threats. Understanding these dual roles of inflammation is important for developing targeted treatments that can mitigate its harmful effects while preserving or even enhancing its beneficial functions. 

## 4. Symptoms of Atherosclerosis

**Figure 1:**
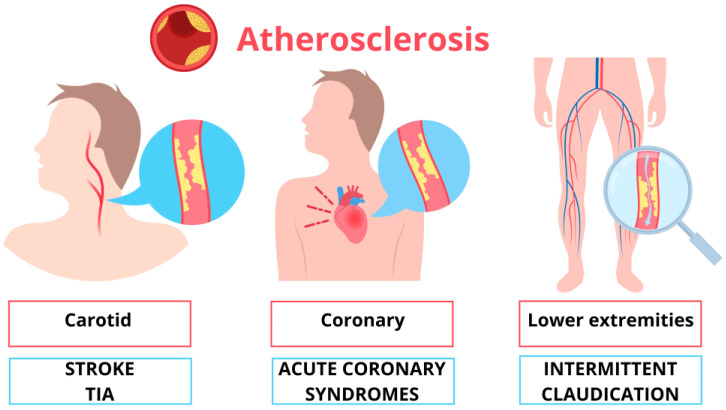
Main sites involved and main symptoms of atherosclerosis.

The excessive concentration of LDL cholesterol and their retention in the intima of the vessels by the extracellular matrix represent the main reason for the accumulation in the intimal layer [75]. In 1977, Goldstein and Brown postulated that the body is designed to maintain LDL cholesterol levels at 25 mg/dL [76,77]. Other evidence suggests that a safe and sufficient level for biological needs could be 20–30 mg/dL [78]. Despite this, LDL levels above 100 mg/dL are frequent in humans, especially in Western countries. The high-fat diet promotes the process of cholesterol accumulation in the blood vessels, triggering atherosclerosis. The 2021 ESC guidelines on cardiovascular prevention [15] recommend using new algorithms for CV risk evaluation [79]. In defining the risk, clinical and laboratory parameters are considered [79]. For each of the above categories, different risk profiles have been identified (low-moderate, high, and very high) based on the probability of developing fatal and non-fatal cardiovascular diseases over ten years, allowing the identification of blood tests, blood pressure, and LDL cholesterol goals. A patient at very high risk is advised to maintain LDL cholesterol < 1.4 mmol/L (less than 55 mg/dL) and a reduction of ≥50% from baseline [79]. For patients with atherosclerotic cardiovascular disease who experience a second event within two years despite maximally tolerated statin therapy, a target LDL level < 1 mmol/L (<40 mg/dL) may be considered [79]. Oxidized LDL promotes atherosclerosis, contributing to the formation of foam cells. Macrophages internalize LDL particles through scavenger receptors, inducing inflammation and immune response [80,81,82]. Cholesterol deposition and inflammatory signaling contribute to plaque growth, which causes narrowing in the vessel lumen, arterial stenosis, and vessel fragility. Various studies show that the thickness of the fibrous cap, calcification, inflammation, infiltration, and the lipid core determine plaque instability. An acute cardiovascular complication of atherosclerosis results from a complex process involving unbalanced opposing forces: pro-thrombotic factors and factors resistant to thrombosis. If the prothrombotic environment prevails during plaque erosion or rupture, an acute ischemic event occurs; otherwise, if antithrombotic factors contain the formation of thrombi, plaque healing occurs [83]. Atherosclerosis begins in childhood, as demonstrated by autopsy studies conducted on children and young adults who died from extra-cardiovascular causes (non-cardiac problems). In the Bogalusa Heart Study, autopsies performed on 204 young subjects (average age 19.6 years) have demonstrated fatty streaks in half of cases aged up to 15 years and in 85% of subjects aged between 21 and 39 years [84,85]. The prevalence of fibrous plaques detected in the aorta and coronary arteries also increases with age, from approximately 20% in subjects aged 2 to 15 to 70% in those aged 26 to 39 [84]. The main arteries affected by atherosclerosis are the aorta, coronary arteries, carotid and cerebral arteries, and renal and peripheral arteries [86]. In each location, the symptoms and complications related to atherosclerosis can be acute, due to the formation of thrombi, or chronic, due to ischemia secondary to the narrowing of the lumen (Figure 1). Typical symptoms are angina, cold sweats, dizziness, extreme tiredness, palpitations, shortness of breath, nausea and weakness, and even erectile dysfunction. When atherosclerosis affects the coronary arteries, it can cause acute coronary syndromes, including myocardial infarction or chronic conditions, such as angina pectoris; both present as chest pain and discomfort caused by insufficient perfusion of the heart muscle due to ischemia [87]. Atherosclerosis involving cerebral vessels causes ischemic stroke or transient cerebral ischemic attack (TIA). Carotid atherosclerosis is related to brain damage and cerebrovascular diseases through different mechanisms: flow reduction, vascular reserve and stiffness alteration, and embolism. Increased intima-media thickness lowers intracranial blood pressure and reduces blood flow velocity by narrowing the vessel lumen. Furthermore, the synergistic effect of increased carotid IMT and carotid artery stiffness leads to less cerebral autoregulation [88,89], increasing the amount of white matter changes [90]. Carotid atherosclerosis might be associated primarily with TIA or stroke as acute consequences of a sudden reduction or interruption of blood flow. Still, it also plays a role in the development of dementia and cognitive impairment. Conventionally, asymptomatic carotid atherosclerotic disease refers to atherosclerotic narrowing in the absence of ipsilateral episodes of TIA or stroke in the preceding six months [91]. Symptomatic carotid artery disease is defined as focal and specific neurologic symptoms in the vascular distribution of a stenotic carotid artery. Atherosclerosis compromises the rigidity and elasticity of the vessels; this can lead to the formation of aneurysms. When it affects the peripheral arteries, it can cause intermittent claudication, ulceration, and necrosis, compromising the integrity of the limbs. 

## 5. Carotid Atherosclerosis

### 5.1. Epidemiology and Clinical Relevance

Two main pairs of arteries ensure cerebral perfusion: the internal carotid arteries for the anterior segments and the vertebral arteries for the posterior ones. The internal carotid artery arises from the common carotid artery and enters the skull. Typically, the plaque initially develops in the carotid bifurcation and proximal internal carotid artery, usually being most severe within 2 cm of the bifurcation of the common carotid artery and predominantly involving the posterior wall of the vessel, developing an hourglass stenotic configuration over time. Regardless of their location, carotid plaques were associated with an increased risk of stroke [92] and an increased risk of mortality in observational studies of elderly adult men [93]. From a review of 59 articles from 21 different countries on the global prevalence of carotid atherosclerosis, Song P. et al. highlighted that the prevalence of carotid atherosclerosis in the world represents 28% of carotid IMT, 21% of plaque, and 1.5% of stenosis > 50% among subjects aged 30 to 79 years [94]. The incidence of carotid atherosclerosis increases globally with age, and women have a lower prevalence than men. Due to ageing over the past twenty years, the prevalence of carotid atherosclerosis increased in IMT by 57.49%, plaque by 58.97%, and stenosis by 59.13%, with the subject group aged between 50 and 55 years [95,96]. In a meta-analysis of over 23,000 people conducted in 2010, the prevalence of asymptomatic severe carotid stenosis, defined as more than 70% narrowing, in the general population ranged from 0 to 3.1%, increasing with age and in the male gender [94,97]. To our knowledge, carotid IMT increases nearly threefold between ages 20 and 90. Postmortem studies indicate that age-associated increases in carotid wall thickening are primarily caused by intimal thickening [98]. Furthermore, increased carotid IMT and localization of carotid IMT are independent and significant predictors of cerebrovascular and cardiovascular diseases [99,100]. One study showed that an increase of 0.1 mm was associated with a 15% increased risk of acute myocardial infarction and an 18% increased risk of stroke [100]. Increased IMT in the internal carotid arteries is more predictive of stroke risk than IMT in the common carotid artery [101].

### 5.2. Stroke and TIA

The mechanisms of ischemic events mediated by carotid artery atherosclerosis generally differ from acute coronary syndromes, as carotid arteries have a much larger diameter and different hemodynamic conditions. Histopathological, epidemiological, and clinical studies show that unstable plaques in the carotid arteries are more likely to cause symptoms, regardless of the severity of the stenosis [102]. In addition to the reduction in the vessel lumen diameter induced by the increase in plaque, thrombi can be deposited on the atheroma, increasing the degree of stenosis. Therefore, the stroke mechanism may be thrombotic material embolism or low flow due to stenosis with inadequate collateral compensation [102]. TIAs can also be due to reduced flow or embolization. Low-flow TIAs without adequate collateral blood supply are short, repetitive episodes [103]. 

In contrast, embolic TIAs are usually single and more prolonged, with symptoms related to the involved vascular territories, usually the middle cerebral or even the anterior cerebral artery. When the internal carotid artery becomes completely occluded, it can also cause low-flow ischemic or embolic events, depending on the adequacy of collateral flow through the circle of Willis. Furlan AJ et al. studied a delayed stroke phenomenon, which occurs many months after carotid occlusion, presumably due to the propagation of a thrombus or embolism from the distal portion of the clot [104]. Impaired cerebral hemodynamic function may be an essential factor in the occurrence of symptoms and stroke in patients with carotid stenosis. The prognosis of patients with stroke due to carotid occlusion may be related to collateral flow [103].

Furthermore, symptomatic patients have a more compromised cerebrovascular reserve than asymptomatic ones [103]. Impaired vasoreactivity of cerebral arteries increases the risk of ipsilateral ischemic stroke in patients with severe carotid stenosis or occlusion [103]. The risk was similar when comparing subgroups with symptomatic versus asymptomatic carotid stenosis.

### 5.3. Cognitive Impairment/Dementia

The baseline level of IMT is a good independent predictor of new cognitive impairment in middle-aged patients. Among patients without stroke, vulnerable plaque and the degree of carotid stenosis had a strong relationship with decreased cognitive function [105,106]. Patients with severe carotid stenosis always had a lower score on the mini-mental state exam than the group with mild to moderate carotid stenosis (40–70%) [107]. Another possible mechanism included in the development of dementia could be reduced neuronal viability due to chronic hypoperfusion of the brain [107]. This correlates with poorer cognitive performance in patients with asymptomatic carotid stenosis [107]. Carotid atherosclerosis is strongly associated with cerebral small vessel disease, and the pathological pathway could be attributed to microembolism and shared risk factors. Vulnerable plaques are more prone to microvascular changes and embolization, causing silent microinfarctions and strokes. Vulnerable internal carotid artery plaques strongly correlate with the amount of white matter hyperintensity detected by brain magnetic resonance imaging (MRI) [108].

### 5.4. Carotid Atherosclerosis as a Mirror of Systemic Cardiovascular Disease

Atherosclerosis is a systemic disease. Carotid IMT and smooth muscle hypertrophy and hyperplasia are considered the initial stages of systemic atherosclerosis. A strong correlation between carotid IMT and the ABI (Ankle-Brachial Index), a non-invasive index of peripheral artery disease, has emerged from previous evidence [109], as in the case of coronary atherosclerosis with stenosis > 50% assessed with coronary angiography and with intravascular ultrasound (IVUS) [110]. Furthermore, carotid IMT and plaque score strongly correlate with the Syntax Score, an angiographic score of coronary plaque burden [111] and the number of coronary vessels involved [112]. Carotid atherosclerosis is also associated with renal artery stenosis and aortic stenosis [113].

## 6. Prevention Strategies and Clinical Practice: What Is the Future?

### 6.1. Cardiovascular Risk Factors

**Figure 2:**
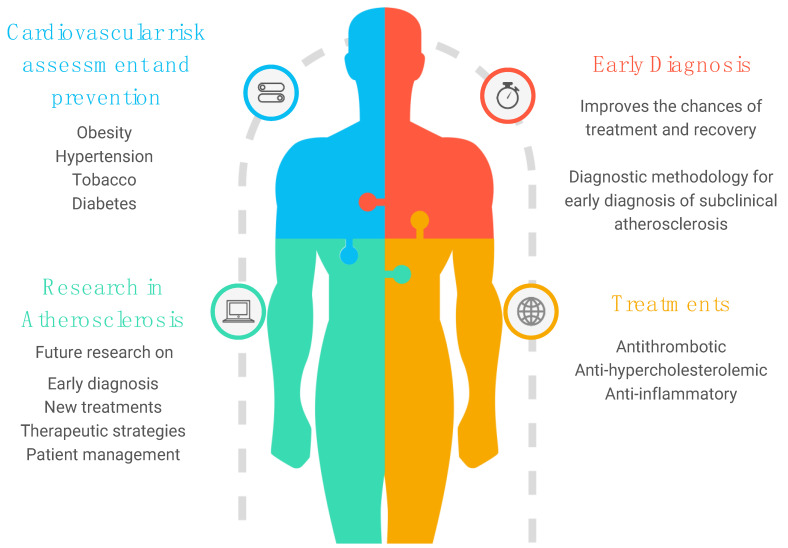
Prevention strategies in the context of atherosclerosis.

Therapeutic improvements alone cannot address the enormous global burden of cardiovascular diseases and their complications. Therefore, prevention strategies in the context of atherosclerosis must be planned (Figure 2). Carotid atherosclerosis can lead to ischemic stroke or TIA, associated with an annual risk of 0.5 to 1.0% in patients with asymptomatic carotid atherosclerosis [113]. Asymptomatic carotid atherosclerosis is also an indicator of increased risk of myocardial infarction and vascular death [113]. Therefore, this condition is considered an equivalent risk for cardiovascular disease. First, lifestyle modification should always be supported as a starting point for prevention. A healthy lifestyle includes physical activity and diet changes, as recommended by the 2021 ESC guidelines on cardiovascular prevention [79]. Obesity is a pandemic of our century that enormously affects the health of populations, as does the use of tobacco and a sedentary lifestyle in young people and adults. The Mediterranean diet determines positive effects on reducing cardiovascular events in patients with carotid artery atherosclerosis [114]. The use of tobacco should be strongly discouraged because its cessation is probably the only significant measure of prevention of atherosclerosis, and that of the carotid artery is no exception [95]. Another crucial objective of the prevention strategy is risk factors. There is a strong correlation between exposure to them in the early stages of life and future cardiovascular events [115] and cognitive deterioration. Hypertension in young people increases the risk of carotid atherosclerosis compared to onset in adulthood [116].

In contrast, well-controlled hypertension in childhood is associated with a markedly reduced risk [116]. Treatment of hypertension in asymptomatic patients with carotid atherosclerosis has demonstrated a concrete reduction in stroke and cardiovascular risk [117]. The association between diabetes and worsening glycemic control and carotid plaques is well known. Carotid plaques were found in 64.2% of patients with diabetes versus 34.2% in controls [118]. Furthermore, diabetes increases the risk of death after carotid endarterectomy (CEA) 4-fold in patients with carotid atherosclerosis [119].

### 6.2. Anti-Hypercholesterolemic Treatment

A meta-analysis of 17 studies reporting 5-year mortality in patients with asymptomatic carotid atherosclerosis highlighted the potential importance of aggressive statin therapy [120]. Furthermore, in the acute setting of TIA, administration of a statin has been reported to result in a significant decrease in stroke at 7 days [121]. General treatment with a statin reduces mortality and cardiovascular events in patients with CEA [122]. After only one month of statin treatment, a favorable change in plaque morphology occurs with decreased plaque progression, remodeling, and even plaque regression, especially when LDL-C was maintained at <100 mg/dL [123]. This highlights the need for a markedly reduced LDL-C level to prevent high-risk cardiovascular diseases, including carotid plaque. Table 1 summarizes treatment goals and some key interventions for different patient categories. The pharmacological approach involves a stepwise strategy to achieve the defined LDL target based on the category according to the CV risk score [79]. The same guidelines provide the absolute reductions in LDL levels that can be achieved with different therapeutic approaches. The estimated mean decrease in LDL level ranges from 30% with moderate-intensity statin therapy to 50% with high-intensity statin therapy and up to 65% with the addition of ezetimibe. Adding a PCSK9 inhibitor to high-intensity statin therapy alone or combined with ezetimibe can reduce LDL levels of 75% and 85%, respectively. The effect obtained with a high-intensity statin can be achieved by administering moderate-intensity statins + ezetimibe [124].

### 6.3. Antithrombotic Treatment in Carotid Artery Atherosclerosis: From Certainties to Dilemma

Besides the careful control of CV-risk factors, antithrombotic agents, particularly aspirin, may be considered for secondary prevention and primary prevention in patients at high cardiovascular risk. Even though several pieces of evidence have confirmed the association between carotid artery atherosclerosis and CV events, this condition is not universally recognized as an independent risk. In this field, the decision to prescribe aspirin in the context of primary prevention should be based on something other than an individual’s absolute CV risk assessment for future events. However, even if broad agreement exists on the use of aspirin in secondary prevention, there is substantial controversy about its use in primary prevention [125]. The 2021 ESC guidelines on CV prevention strongly recommend aspirin for secondary prevention of CV disease and suggest it in patients with diabetes mellitus at high or very high CV risk for primary prevention without clear contraindications [79]. In 2009, a meta-analysis of patients with low CVD risk reported a 12% reduction in ASCVD with aspirin but a significant increase in major bleeding [125]. An updated meta-analysis did not show a reduction in all-cause or CV mortality with aspirin. Still, it did show a lower risk of non-fatal myocardial infarction (RR 0.82, 95% CI: 0.72 to 0.94) and ischemic stroke (RR 0.87, 95% CI: 0.79 to 0.95) [126]. Conversely, aspirin was associated with a higher risk of major bleeding (RR 1.43, 95% CI: 1.30 to 1.56), intracranial bleeding (RR 1.34, 95% CI: 1.14 to 1.57), and major gastrointestinal bleeding (RR 1.56, 95% CI: 1.38 to 1.78) [127]. In non-cardioembolic ischemic stroke, aspirin is the most studied antithrombotic drug. Aspirin reduces the risk of recurrent ischemic stroke and vascular severe events [79]. In patients with ischemic stroke or TIA, prevention with antithrombotic is recommended. The use of an antiplatelet is recommended for patients with non-cardioembolic ischemic stroke or TIA, and the use of an anticoagulant is recommended in patients with cardioembolic ischemic stroke or TIA [79]. Wider use than this could amplify the benefit of aspirin in primary prevention for patients with higher atherosclerotic risk. It is reasonable to consider treatment with aspirin in patients assessed for high and very high cardiovascular risk according to the SCORE2 score, always taking into account the risk of bleeding [125,127]. We know that the presence of carotid plaque has a strong predictive risk for MACE and could be a valuable tool to reclassify the 8–15% of patients at intermediate cardiovascular risk into a higher risk class [128]. Furthermore, from the Atherosclerosis Risk in Communities (ARIC) study, it emerges that the addition of carotid atherosclerosis to the traditional risk factors reclassifies 39.4% of the population at intermediate risk (10–20% risk at ten years) into a different CV risk category, with a significant overall improvement in CV risk prediction [129]. While the efficacy of antiplatelet agents in patients with symptomatic carotid atherosclerosis is well evidence-based, few data are available for asymptomatic carotid atherosclerosis. The Asymptomatic Cerebral Bruit (ACB) study showed no differences in annual MACEs between patients with asymptomatic carotid stenosis treated with aspirin and those in the placebo group [130]. In contrast, in an observational study evaluating the effect of optimal medical therapy (statins, antihypertensive agents, and antiplatelet agents) on cerebrovascular events in a small cohort of patients with high-grade (≥70%) asymptomatic carotid stenosis, only antiplatelet agents were independent predictors of significantly reduced risk of ipsilateral stroke/TIA and any stroke and cardiovascular death at two years [117]. One of the certainties about antiplatelet drugs in carotid atherosclerosis is their effect on reducing plaque size; according to a meta-analysis of 47 studies, the average annual reduction is 0.033 mm [131]. Finally, the finding of asymptomatic carotid atherosclerosis cannot be considered a direct indication for the use of antithrombotic drugs, and the choice to use aspirin or new antithrombotic regimens should be made only after a careful estimate of the cardiovascular and hemorrhagic risk. Indeed, the degree of stenosis, the complexity of the plaques and the involvement of other vascular beds, all associated with an increased cardiovascular risk, firmly shift the balance in favor of the benefit of antithrombotic prophylaxis.

### 6.4. Anti-Inflammatory Treatment

Presently, two drugs have demonstrated efficacy in reducing the inflammatory component of atherosclerosis during phase III clinical trials: Canakinumab and Colchicine. The CANTOS trial tested the subcutaneous administration every three months of Canakinumab, an anti-interleukin-1β antibody, versus placebo in 10,061 patients with previous MI and high-sensitivity C-reactive protein (hsCRP) levels of ≥2.0 mg/L. At a median follow-up of 3.7 years, the results revealed a notable 15% reduction in the primary endpoint (non-fatal myocardial infarction, any non-fatal stroke, or CVD) with administration of 150 mg of Canakinumab regardless of lipid-level lowering [132]. Colchicine is an oral drug that inhibits tubulin polymerization, blocking the cytoskeletal microtubule formation. Colchicine is commonly used in pericarditis, Mediterranean fever, and acute gout. Recently, Colchicine, with an oral administration of 0.5 mg once daily, was tested in two trials performed, respectively, in 5522 CCS patients (LoDoCo2) and in 4745 post-MI within 30 days subjects (COLCOT) [133,134]. In LoDoCo2, at a median follow-up of 28.6 months, Colchicine showed a 31% reduction in primary endpoint (CVD, spontaneous nonprocedural MI, ischemic stroke, ischemia-driven coronary revascularization) compared to placebo [133]. In COLCOT, at a median follow-up of 22.6 months, Colchicine demonstrated a 23% reduction in primary endpoint (CVD, resuscitated cardiac arrest, MI, stroke, urgent hospitalization for angina leading to coronary revascularization) [134]. In addition to Canakinumab and Colchicine, other immunotherapies, like Tocilizumab, an anti-interleukin-6 receptor, in the ASSAIL-MI trial, and Anakinra, an IL-1 receptor antagonist, in the MRC-ILA trial, showed potential benefits during phase II trials [135,136]. However, despite the successes observed in trials such as CANTOS, ASSAIL-MI, and MRC-ILA, several drawbacks were noted for most of these drugs, including high costs, adverse effects, such as sepsis and pneumonia, and administration routes. Among the mentioned drugs, only Colchicine stands out for its relatively lower cost and oral administration, demonstrating a favorable impact on cardiovascular events and thus approved by the FDA for cardiovascular prevention. Nevertheless, its use remains limited in clinical practice due to the results of some studies, such as the COPS trial, in which Colchicine did not significantly affect cardiovascular outcome at 1 year in ACS subjects, being further associated with a higher non-cardiovascular mortality, primarily associated with the onset of serious infections [137]. However, currently several trials, such as ZEUS (NCT05021835) and PULSE-MI (NCT05462730), targeting inflammation in the context of cardiovascular diseases, are ongoing with initial results expected in the coming years.

## 7. Conclusions

Atherosclerosis afflicts the global population with its clinical implications and various cardiovascular events, such as myocardial infarction, stroke, and peripheral arterial disease. This comprehensive review highlights the significant role of atherosclerosis in cardiovascular disease (CVD), underlining its systemic and chronic nature. Starting from an overview of the progression of the disease from asymptomatic stages to advanced manifestations, it explores the crucial role of inflammation in atherogenesis, clarifying the complex interaction between endothelial dysfunction, lipid accumulation, and immune response. Furthermore, carotid atherosclerosis is related to stroke and TIA but also cognitive impairment and dementia. Adequate cardiovascular prevention and clinical management approaches, lifestyle modifications, and risk factor control are essential to reduce the impact of this complex disease. The role of hypocholesterolemic and antithrombotic therapy is also fundamental in primary and secondary prevention. In conclusion, this review provides a comprehensive understanding of atherosclerosis. It highlights the importance of early diagnosis, risk stratification, and targeted interventions to mitigate the burden of cardiovascular disease associated with atherosclerosis.

## Figures and Tables

**Table 1:** Treatment goals for different patient categories.

Patient Category	Prevention Goals (STEP 1)	Intensified/Additional Prevention Goals (STEP 2)
Apparently healthy persons	Stop smoking and lifestyle SBP < 140 to 130 mmHg LDL-C < 2.6 mmol/L (100 mg/dL)	SBP < 130 mmHg LDL-C < 1.8 mmol/L (70 mg/dL) and >50% reduction in high-risk patients LDL-C < 1.4 mmol/L (55 mg/dL) and >50% reduction in very-high-risk patients
Patients with CKD	Stop smoking and lifestyle optimization SBP < 140 down to 130 mmHg LDL-C < 2.6 mmol/L (100 mg/dL) and >50% LDL-C reduction Otherwise according to ASCVD and DM history	LDL-C < 1.8 mmol/L (70 mg/dL) in high-risk patients and <1.4 mmol/L (55 mg/dL) in very-high risk patients
Patients with FH	Stop smoking and lifestyle optimization SBP < 140 down to 130 mmHg LDL-C < 2.6 mmol/L (100 mg/dL) and >50% LDL-C Reduction. Otherwise according to ASCVD and DM history	LDL-C < 1.8 mmol/L (70 mg/dL) in high-risk patients and <1.4 mmol/L (55 mg/dL) in very-high risk patients
Patients with type 2 DM without established ASCVD or severe TOD	Stop smoking and lifestyle optimization SBP < 140 down to 130 mmHg LDL-C < 2.6 mmol/L (100 mg/dL) HbA1c < 53 mmol/mol (7.0%)	SBP < 130 mmHg LDL-C < 1.8 mmol/L (70 mg/dL) and >50% reduction SGLT2 inhibitor or GLP-1RA
Patients with type 2 DM with established ASCVD or severe TOD	Stop smoking and lifestyle optimization SBP < 140 down to 130 mmHg LDL-C < 1.8 mmol/L (70 mg/dL) HbA1c < 64 mmol/mol (8.0%) SGLT2 inhibitor or GLP1-RA CVD: antiplatelet therapy	SBP < 130 mmHg LDL-C < 1.4 mmol/L (55 mg/dL) and >50% reduction SGLT2 inhibitor or GLP-1RA if not already on May additionally consider novel upcoming treatments: DAPT, dual pathway inhibition, colchicine, icosapent ethyl, etc.
Patients with established ASCVD	Stop smoking and lifestyle optimization SBP < 140 down to 130 mmHg Intensive oral lipid-lowering therapy aiming at >50% LDL-C reduction and LDL-C < 1.8 mmol/L (70 mg/dL) Antiplatelet therapy	SBP < 130 mmHg LDL-C < 1.4 mmol/L (55 mg/dL) May additionally consider novel upcoming treatments: DAPT, dual pathway inhibition, colchicine, icosapent ethyl, etc.

ASCVD = atherosclerotic cardiovascular disease; BP = blood pressure; CKD = chronic kidney disease; CVD = cardiovascular disease; DAPT = dual antiplatelet therapy; DBP = diastolic blood pressure; DM = diabetes mellitus; EAS = European Atherosclerosis Society; ESC = European Society of Cardiology; FH = familial hypercholesterolemia; GLP-1RA = glucagon-like peptide-1 receptor agonist; HbA1c = glycated hemoglobin; LDL-C = low-density lipoprotein cholesterol; SBP = systolic blood pressure (office); SGLT2 = sodium-glucose cotransporter 2; TOD = target organ damage.
